# Complete lymph node dissection versus selective lymph node extirpation in melanoma patients with nodal macrometastasis and adjuvant systemic therapy

**DOI:** 10.1111/ddg.15967

**Published:** 2026-01-21

**Authors:** Kristine E. Mayer, Jessica C. Hassel, Jannik Sambale, Michael Erdmann, Mihaela‐Anca Sindrilaru, Julia Oberschmied, Alexander Thiem, Dirk Tomsitz, Jana Knuever, Max Schlaak, Carola Berking, Christian Posch, Tilo Biedermann, Oana‐Diana Persa

**Affiliations:** ^1^ Department of Dermatology and Allergy School of Medicine Technical University of Munich (TUM) Bavarian Center for Cancer Research (BZKF) Munich Germany; ^2^ Department of Dermatology and National Center for Tumor Diseases (NCT) NCT Heidelberg Medical Faculty Heidelberg Heidelberg University a partnership between DKFZ and University Hospital Heidelberg Heidelberg Germany; ^3^ Department of Dermatology Uniklinikum Erlangen Comprehensive Cancer Center Erlangen ‐ EMN Friedrich‐Alexander‐Universität Erlangen‐Nürnberg (FAU) CCC Alliance WERA Bavarian Center for Cancer Research (BZKF) Erlangen Germany; ^4^ Department of Dermatology and Allergology Universitätsklinikum Ulm Ulm Germany; ^5^ Department of Dermatology Venereology and Allergology Charité – Universitätsmedizin Berlin Berlin Germany; ^6^ Department of Dermatology and Venerology Universitätsmedizin Rostock Rostock Germany; ^7^ Department of Dermatology and Allergy LMU University Hospital LMU Munich Munich Germany; ^8^ Department of Dermatology and Venerology Universitätsklinikum Köln Cologne Germany; ^9^ Centre for Skin Diseases Department of Dermatology and Allergy University Hospital Bonn Bonn Germany; ^10^ Faculty of Medicine Sigmund Freud University Vienna Austria; ^11^ Klinik Hietzing Wiener Gesundheitsverbund Vienna Austria

**Keywords:** Adjuvant therapy, lymph node dissection, lymph node extirpation, lymph node metastasis, macrometastasis, melanoma

## Abstract

**Background and Objectives:**

Complete lymph node dissection (CLND) is the standard of care in patients with regional nodal melanoma macrometastasis. However, evidence on surgical procedures in the era of adjuvant systemic therapies is lacking.

**Patients and Methods:**

This retrospective multi‐center study included stage IIIB–D melanoma patients with nodal macrometastasis undergoing CLND or selective lymph node extirpation (LNE) prior to adjuvant therapy. CLND and LNE were compared regarding recurrence‐free survival (RFS), nodal metastasis‐free survival (NFS) and overall survival (OS).

**Results:**

320 melanoma patients were included (median age 62; 55.5% male). Patients received PD‐1 monotherapy (77.8%), targeted therapy (21.3%) or both sequentially (0.9%), as well as adjuvant radiotherapy in 40.9%. RFS and OS did not differ significantly between patients receiving CLND or LNE, while NFS was significantly prolonged following CLND (HR 0.3917; p = 0.005). After adjustment for risk factors by multivariate Cox regression, a prolonged RFS for CLND vs. LNE was found (HR 0.676; p = 0.04), but no benefit for OS. The rate of complications was significantly higher in the CLND group.

**Conclusions:**

CLND showed no OS benefit compared to LNE, while local control was improved. CLND can be recommended in the context of adjuvant therapies, however, the increased rate of complications should be considered.

## INTRODUCTION

Patients with thick primary melanomas or regional metastases have a high risk for disease recurrence after surgical resection that varies between 35% for stage IIB and 79% for stage IIID.^1^ Therefore, patients are typically offered adjuvant therapy, either with immune checkpoint inhibitors (ICIs) targeting PD‐1^2^, ^3^, ^4^ or, in the case of an activating BRAFV600 mutation and stage III disease, a combination of BRAF and MEK inhibitors.^5^, ^6^ The adjuvant therapies have a similar efficacy in reducing the relative risk for recurrence with a hazard ratio of 0.61 for anti‐PD‐1,^2^ and of 0.51 for targeted therapy.^5^ Patients with stage III disease included in the respective clinical trials all received complete lymph node dissection (CLND) as inclusion criteria per protocol. Hence, to date it is unknown if patients receiving adjuvant therapy still benefit from CLND or would have similar clinical outcomes with extirpation of the lymph node metastases (LNE) only. Lately, neoadjuvant treatment concepts have shown excellent rates of major pathologic response, and event‐free survival upon neoadjuvant treatment with the combination of ipilimumab and nivolumab or monotherapy with pembrolizumab. This questions the role of CLND followed by adjuvant treatment in a subgroup of patients with stage III melanoma.^7^, ^8^, ^9^ However, while most patients profit from a neoadjuvant treatment strategy, there is a subgroup of patients that progress under neoadjuvant treatment, suggesting that CLND could still play a role in the treatment of stage III patients in the future.

In patients with micrometastasis in their sentinel lymph node biopsy (SLNB), CLND is no longer recommended, as randomized studies have shown that CLND neither prevents recurrence nor distant metastasis and had no significant impact on overall survival (OS).^10^, ^11^, ^12^ These findings are supported by further retrospective analyses.^13^, ^14^, ^15^, ^16^, ^17^ Even in patients with multiple positive sentinel lymph nodes (SLN) CLND did not significantly improve melanoma‐specific survival or recurrence‐free survival (RFS).^17^

In patients with lymph node macrometastasis, however, there is a lack of evidence regarding the surgical management of patients. In the era before adjuvant PD‐1 and BRAF/MEK inhibitor treatment, CLND was commonly performed due to missing alternatives.^18^, ^19^, ^20^ Several studies on patients receiving CLND identified clinical parameters influencing RFS and OS such as the number of lymph nodes involved and the thickness of the primary tumor.^18^, ^21^ Prior to the introduction of novel adjuvant therapies, a higher number of excised lymph nodes was described to be associated with a better prognosis in stage III patients.^22^, ^23^ In the cohort of Rossi and colleagues, which included 2507 stage III patients, the median number of excised lymph nodes was 15, depending on the anatomical location.^22^ Multivariate subgroup analysis revealed that patients with micrometastasis, but not those with macrometastasis, benefited from a larger number of excised lymph nodes.^22^ Additionally, a prognostic benefit from a larger number of removed lymph nodes was observed only in patients with two to three positive lymph nodes.^22^ In the context of effective adjuvant systemic therapies, it is therefore essential to determine whether patients with lymph node macrometastasis still benefit from additional regional CLND, or whether LNE leads to an equivalent clinical outcome.

Besides its prognostic implications, CLND is associated with higher morbidity.^24^ In lymph node surgery, long‐lasting complications, such as lymphedema or paresis resulting from nerve damage, must be taken into consideration. Previous studies have shown that a larger number of excised lymph nodes correlates with more postoperative complications.^25^ If there is no survival benefit as observed in a comparison of ilio‐inguinal vs. inguinal lymphadenectomy,^26^, ^27^ CLND should be avoided.

This retrospective multi‐center study therefore assessed the clinical outcome after CLND and LNE in patients with lymph node macrometastasis in the era of modern adjuvant therapies. It aimed to support decision making in the daily clinical routine by providing evidence on possible benefits and/or disadvantages of the two interventions.

## MATERIAL AND METHODS

### Study design

Melanoma patients of AJCCv8 stage IIIB–IIID who underwent either CLND or LNE between 2015 and 2024 were identified retrospectively from eight participating university hospitals with specialization in dermato‐oncology in Germany. The study was approved by the ethical committee of the Technical University of Munich. Inclusion criteria were histologically confirmed diagnosis of melanoma, clinically or radiographically suspected lymph node macrometastasis, absence of distant metastasis at intervention, performance of either CLND or LNE, histologic confirmation of at least one lymph node metastasis, and adjuvant systemic treatment with a PD‐1 inhibitor, BRAF/MEK inhibition or both sequentially. LNE was defined as the surgical excision of pre‐ and intraoperatively clinically suspect lymph nodes only. CLND summarizes systematic inguinal, axillary, or cervical lymphadenectomy.

In addition to patient and tumor characteristics, treatment regimens, and the following parameters were collected: number of lymph nodes resected, number of histologically confirmed lymph node metastases, diameter of the largest lymph node metastasis, presence of extracapsular spread, performance of adjuvant radiation therapy, performance and duration of adjuvant systemic therapy, side effects associated with the surgical interventions, occurrence of relapse or death and corresponding time from the intervention, type of progression, as well as length of the follow‐up period. The primary objective of the study was to evaluate the impact of CLND or selective LNE in patients with lymph node macrometastasis on RFS, nodal metastasis‐free survival (NFS) and OS. RFS was defined as the time from intervention (CLND/LNE) to the occurrence of relapse, while NFS was defined as the time from the intervention to the detection of a locoregional lymph node metastasis. OS was defined as the time from the intervention to death. Patients without an event were censored at the last patient contact.

### Statistical analysis

GraphPad Prism v10.4.0 and IBM SPSS Statistics v29.0.2 were used to visualize the results and to perform statistical tests. To compare survival benefits between the groups, two‐sided log‐rank tests of Kaplan‐Meier curves were used, and median RFS, NFS and OS were determined. The median follow‐up was calculated as the duration from intervention (CLND/LNE) to death or the last patient contact.

The unadjusted risk estimates of different baseline parameters including the interventions on RFS and OS were assessed by univariate Cox proportional hazards regression analysis. The baseline parameters included sex, age, tumor thickness, ulceration, BRAF mutation, lactate‐dehydrogenase (LDH) level (elevated vs. not elevated) at the start of adjuvant therapy, number of resected lymph nodes, more than three metastatic lymph nodes (yes vs. no), diameter of the largest lymph node metastasis, extracapsular spread (yes vs. no), surgical intervention (CNLD vs. LNE), adjuvant radiation therapy (yes vs. no), adjuvant systemic therapy (BRAF/MEK inhibition vs. PD‐1 inhibitor), and duration of adjuvant systemic therapy in months.

RFS and OS were evaluated using a time‐dependent Cox proportional hazards multivariate model stratified by more than three metastatic lymph nodes or age, and the duration of adjuvant systemic therapy, including the intervention (CLND vs. LNE) as a factor. Hazard ratios (HR) and associated 95% confidence interval (95% CI) are reported from this model; HR and p values were adjusted for presence of all factors. For all analyses, the threshold for statistical significance was set at p < 0.05.

## RESULTS

### Patient characteristics

**FIGURE 1:**
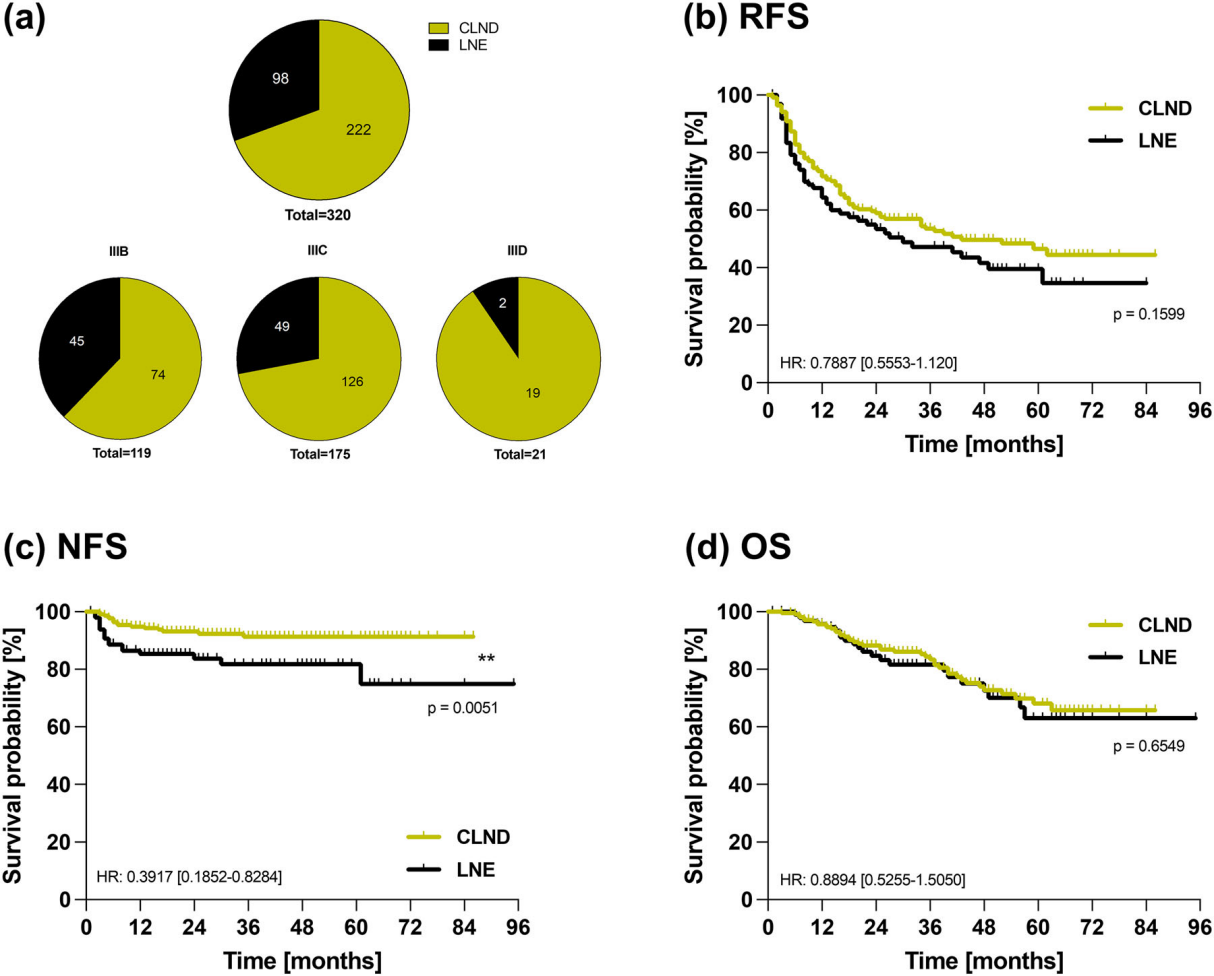
Distribution of disease stages and survival for the entire study cohort after CLND and LNE. (a) Distribution of patients who underwent CLND vs. LNE for the whole study cohort and according to AJCCv8 stages IIIB, IIIC, and IIID; (b) Kaplan‐Meier curves representing recurrence‐free survival (RFS), (c) nodal metastasis‐free survival (NFS), and (d) overall survival (OS) for the overall study cohort according to the intervention (CLND vs. LNE). Hazard ratios (HR) including their 95% confidence interval [95% CI] are shown in the lower left corner of each graph.

A total of 320 patients with lymph node macrometastasis (AJCCv8 stage IIIB–IIID) undergoing CLND or LNE were included. Of these, 222 (69.4%) patients received CLND, while 98 (30.6%) patients underwent LNE. For detailed information see Table 1. The two groups were highly heterogenous. The number of histologically confirmed lymph node metastases was higher in the CLND group. Respectively, differences between the two groups were also observed in terms of the distribution of disease stages according to AJCCv8. Patients later diagnosed with stage IIIC and IIID disease underwent CLND more frequently compared to LNE (Figure 1a). In contrast in the LNE group, the proportion of stage IIIB patients was higher and only two patients were classified as stage IIID (Table 1). The median follow‐up time was 34 months for the CLND group and 37 months for the LNE group.

**TABLE 1:** Patient characteristics of the overall study cohort. Baseline characteristics of the study cohort (N = 320): complete lymph node dissection (CLND) or selective lymph node extirpation (LNE) of suspect lymph nodes. Serum LDH is indicated in the categories normal and elevated, which is defined as above the upper limit of normal (ULN).

	CLND (n = 222; 6.4%)	LNE (n = 98; 30.6%)
***Age (years)***		
Median (Range)	62 (24–88)	64 (28–87)
***Gender***		
Female	90 (40.5%)	53 (54.1%)
Male	132 (59.5%)	45 (45.9%)
***BRAF status***		
V600 wild‐type	107 (48.2%)	59 (60.2%)
V600 mutation	103 (46.4%)	37 (37.8%)
Unknown	12 (5.4%)	2 (2.0%)
***Stage (AJCCv8)***		
IIIB	74 (33.3%)	45 (45.9%)
IIIC	126 (56.8%)	49 (50.0%)
IIID	19 (8.6%)	2 (2.0%)
Unknown	3 (1.4%)	2 (2.0%)
***Tumor thickness [mm]***		
Mean (Range)	4.23 (0.2–17)	3.52 (0.3–11)
***Ulceration of primary***		
Yes	78 (35.1%)	34 (34.7%)
No	109 (49.1%)	42 (42.9%)
Unknown/NA	35 (15.8%)	22 (22.4%)
***Number of removed lymph nodes***		
Mean	14.83	2.55
SD	9.28	4.12
***Number of histologically confirmed lymph node metastases***		
Mean	3.86	1.35
SD	3.87	0.91
***Diameter of largest lymph node metastasis [cm]***		
Mean	2.85	2.45
SD	2.30	1.67
***Extracapsular spread***		
Yes	80 (36.0%)	33 (33.7%)
No	125 (56.3%)	54 (55.1%)
Unknown	17 (7.7%)	11 (11.2%)
***Serum LDH***		
Normal (≤ ULN)	168 (75.7%)	74 (75.5%)
Elevated (> ULN)	33 (14.9%)	15 (15.3%)
Unknown	21 (9.5%)	9 (9.2%)
***Adjuvant systemic therapy***		
PD‐1 inhibitor	167 (75.2%)	82 (83.7%)
BRAF/MEK inhibitor	53 (23.9%)	15 (15.3%)
Both sequentially	2 (0.9%)	1 (1.0%)
***Duration of adjuvant systemic therapy [months]***		
Mean	8.59	8.28
SD	4.40	4.55

*Abbr*.: AJCCv8, American Joint Committee on Cancer, 8th edition; BRAF, B‐Raf proto‐oncogene, serine/threonine kinase; CLND, complete lymph node dissection; LNE, lymph node extirpation; LDH, lactate dehydrogenase; MEK, mitogen‐activated protein kinase kinase; NA, not available; PD‐1, programmed cell death protein 1; SD, standard deviation; ULN, upper limit of normal

### CLND is associated with significantly prolonged NFS, but not RFS and OS in the overall cohort

Despite substantial differences regarding the distribution of disease stages between the CLND and the LNE group, two‐sided log‐rank tests did not show statistically significant differences for RFS (HR 0.7887 [0.5553–1.120], p = 0.16) and OS (HR 0.8894 [0.5255–1.5050], p = 0.65) between the two groups (Figure 1b, d). NFS, however, was significantly prolonged in the CLND group compared to the LNE group (HR 0.3917 [0.1852–0.8284], p = 0.005; Figure 1c). Median RFS was 43 months in the CLND group and 30 months in the LNE group (online supplementary Table S1). Median NFS and OS were not reached in both groups (online supplementary Table S1).

When analyzing the type of progression (multiple entries possible), only the control of regional lymphatic metastasis was improved by CLND (16.3% recurrence with lymph node metastases in the CLND group compared with 33.3% in the LNE group; p = 0.006). This is in line with the observed improved NFS (Figure 1c). The rates of progression with distant metastasis, however, were comparable between the two groups (75.5% and 70.6% respectively; p = 0.55). Also, the rates of in‐transit metastasis or local recurrence in the area of the primary tumor were comparable, though with low incidences (11.2% vs. 13.7%; p = 0.43; online supplementary Table S1). Second‐line treatment upon relapse after LNE consisted mainly of systemic therapy (96%), while second‐line CLND was performed in 17% of patients.

### CLND is associated with prolonged RFS after accounting for relevant covariates

Univariate Cox proportional hazards regression analysis was conducted to assess the impact of various clinical characteristics and treatment factors on RFS (Table 2). Age, sex, BRAF mutation status, LDH levels, number of lymph nodes removed, diameter of the largest lymph node metastasis, and type of adjuvant systemic therapy (BRAF/MEK inhibition or PD‐1 inhibitor treatment) did not significantly affect RFS. However, the presence of extracapsular spread, more than three metastatic lymph nodes, adjuvant radiation therapy (which is recommended when risk factors as the ones mentioned beforehand are present), and shorter duration of systemic therapy were all significantly associated with a shorter RFS. To account for the large clinical differences between the CLND and the LNE group, a multivariate regression model was performed adjusting for relevant covariates which had been identified by univariate Cox proportional hazards regression analysis. CLND was associated with a reduced risk of relapse compared to LNE (HR 0.676 [0.468–0.976], p = 0.04; Table 3). Other independent risk factors for progression were more than three metastatic lymph nodes (HR 2.210 [1.492–3.273], p < 0.001) and duration of adjuvant systemic therapy (HR 0.834 [0.805–0.865], p < 0.001).

**TABLE 2:** Univariate Cox proportional hazards regression models for RFS. P values indicating significance are written in italic.

	Univariate Analysis: HR [95% CI]	*p* value
***General characteristics***		
Age [years]	1.002 [0.991–1.014]	0.68
Sex (female vs. male)	1.077 [0.780–1.485]	0.65
Tumor thickness [mm]	1.000 [0.976–1.025]	>0.99
Ulceration (yes vs. no)	1.273 [0.898–1.806]	0.18
BRAF mutation (yes vs. no)	0.878 [0.633–1.219]	0.44
LDH before systemic therapy (elevated vs. normal)	1.012 [0.633–1.617]	0.96
***Lymph node metastasis***		
Number of resected lymph nodes	0.995 [0.978–1.012]	0.54
More than 3 metastatic lymph nodes (yes vs. no)	1.727 [1.209–2.467]	*0.003*
Diameter of largest lymph node metastasis [cm]	1.041 [0.958–1.131]	0.34
Extracapsular spread (yes vs. no)	1.708 [1.216–2.400]	*0.002*
***Therapy***		
Intervention (CLND vs. LNE)	0.793 [0.566–1.110]	0.18
Adjuvant radiation therapy (yes vs. no)	1.470 [1.066–2.028]	*0.02*
Adjuvant systemic therapy (BRAF/MEK vs. PD‐1 inhibitor)	0.723 [0.482–1.086]	0.12
Duration of adjuvant systemic therapy [months]	0.836 [0.807–0.867]	*< 0.001*

*Abbr*.: BRAF, B‐Raf proto‐oncogene, serine/threonine kinase; CI, confidence interval; CLND, complete lymph node dissection; HR, hazard ratio; LDH, lactate dehydrogenase; LNE, lymph node extirpation; MEK, mitogen‐activated protein kinase kinase; PD‐1, programmed cell death protein 1

**TABLE 3:** Multivariate Cox proportional hazards regression model for RFS. *p* values indicating significance are written in italic.

	Multivariate Analysis: HR [95% CI]	*p* value
More than 3 metastatic lymph nodes (yes vs. no)	2.210 [1.492–3.273]	*< 0.001*
Duration of adjuvant systemic therapy [months]	0.834 [0.805–0.865]	*< 0.001*
Intervention (CLND vs. LNE)	0.676 [0.468–0.976]	*0.04*

*Abbr*.: CI, confidence interval; CLND, complete lymph node dissection; HR, hazard ratio; LNE, lymph node extirpation

### Duration of adjuvant systemic therapy, but not surgical intervention influences OS in melanoma patients with nodal macrometastasis

Next, factors influencing OS were analyzed by univariate Cox proportional hazards regression. Higher age, extracapsular spread of lymph node metastasis, adjuvant radiation therapy and shorter duration of adjuvant systemic therapy were associated with poorer OS, while the type of adjuvant systemic therapy (p = 0.054) and the other test parameters did not significantly influence OS (Table 4). Multivariate Cox regression, however, revealed that only the duration of adjuvant systemic therapy had a significant impact on OS. In particular, no benefit of CLND in comparison to LNE was observed regarding OS (Table 5).

**TABLE 4:** Univariate Cox proportional hazards regression models for OS. *p* values indicating significance are written in italic.

	Univariate Analysis: HR [95% CI]	*p* value
***General characteristics***		
Age [years]	1.021 [1.002–1.040]	*0.03*
Sex (female vs. male)	1.023 [0.622–1.680]	0.93
Tumor thickness [mm]	0.998 [0.963–1.034]	0.90
Ulceration (yes vs. no)	1.313 [0.776–2.221]	0.31
BRAF mutation (yes vs. no)	0.609 [0.361–1.028]	0.06
LDH before systemic therapy (elevated vs. normal)	1.139 [0.572–2.268]	0.71
***Lymph node metastasis***		
Number of resected lymph nodes	0.995 [0.970–1.021]	0.71
More than 3 metastatic lymph nodes (yes vs. no)	1.538 [0.891–2.653]	0.12
Diameter of largest lymph node metastasis [cm]	1.045 [0.918–1.191]	0.51
Extracapsular spread (yes vs. no)	1.800 [1.073–3.020]	*0.03*
***Therapy***		
Intervention (CLND vs. LNE)	0.876 [0.523–1.468]	0.62
Adjuvant radiation therapy (yes vs. no)	1.657 [1.014–2.710]	*0.04*
Adjuvant systemic therapy (BRAF/MEK vs. PD‐1 inhibitor)	0.500 [0.247–1.013]	0.054
Duration of adjuvant systemic therapy [months]	0.845 [0.801–0.892]	*< 0.001*

*Abbr*.: BRAF, B‐Raf proto‐oncogene, serine/threonine kinase; CI, confidence interval; CLND, complete lymph node dissection; HR, hazard ratio; LDH, lactate dehydrogenase; LNE, lymph node extirpation; MEK, mitogen‐activated protein kinase kinase; PD‐1, programmed cell death protein 1

**TABLE 5:** Multivariate Cox proportional hazards regression model for OS. *p* values indicating significance are written in italic.

	Multivariate Analysis: HR [95% CI]	*p* value
Age [years]	1.016 [0.997–1.036]	0.09
Duration of adjuvant systemic therapy [months]	0.847 [0.802–0.895]	*< 0.001*
Intervention (CLND vs. LNE)	1.111 [0.659–1.871]	0.69

*Abbr*.: CI, confidence interval; CLND, complete lymph node dissection; HR, hazard ratio; LNE, lymph node extirpation; OS, overall survival

### CLND is associated with more complications

**FIGURE 2:**
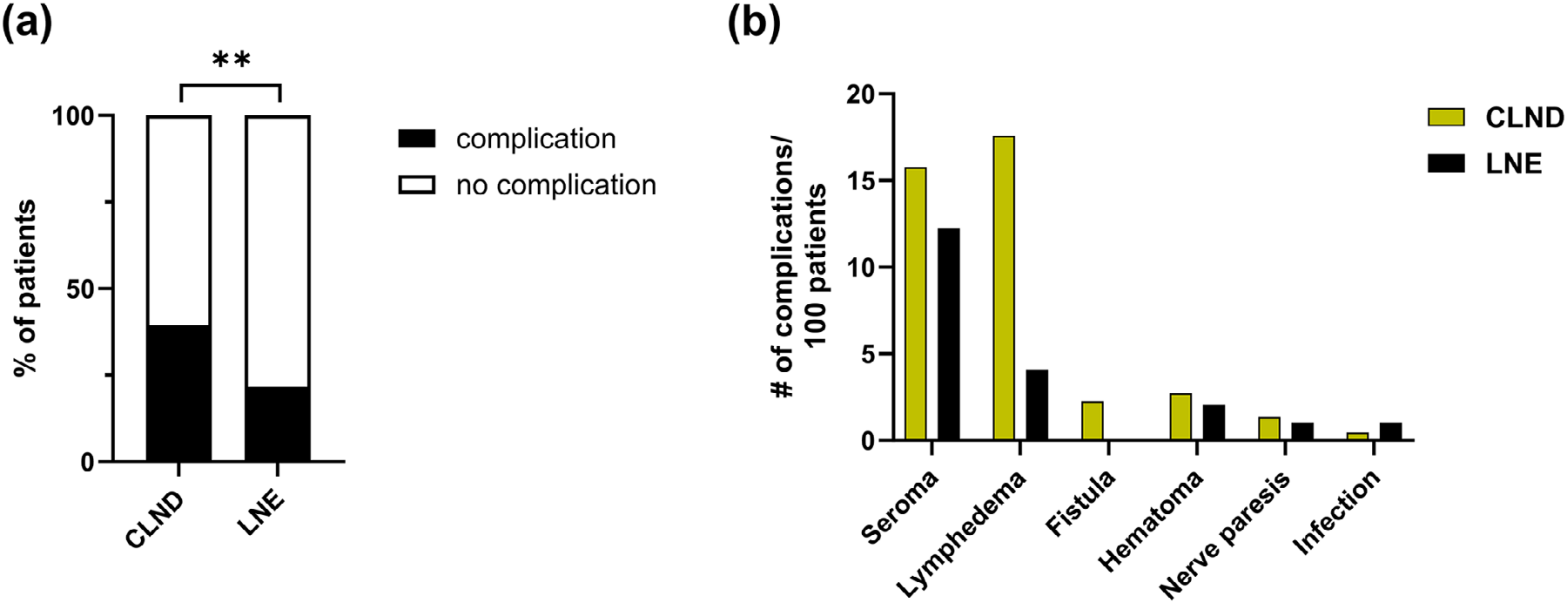
Postoperative complications after CLND and LNE. (a) Percentage of patients experiencing at least one postoperative complication for CLND and LNE; (b) number of complications per 100 patients categorized by type of complication for CLND and LNE.

After revealing benefits on NFS and RFS, but not OS for the CLND group, the risk of complications after each intervention was assessed. The number of patients suffering from at least one postoperative complication was determined. A significantly higher proportion of patients undergoing CLND developed complications compared to those who underwent LNE (Figure 2a). The most common complications reported were seroma and lymphedema (Figure 2b; online supplementary Table S2). While seroma was also a common complication in the LNE group, lymphedema occurred more frequently after CLND. For both interventions, the complication rate varied depending on the localization of the intervention (online supplementary Table S2). After inguinal interventions, the complication rate was significantly higher compared to cervical interventions, for CLND and LNE (Chi‐Square test: p = 0.02 and p = 0.049, respectively). Despite differences in the frequency of complications between different localizations, the causes for complications were similarly distributed within each intervention group (online supplementary Table S2). Overall, fistula formation, hematoma, nerve paresis, and infection were observed in fewer than 5% of patients (Figure 2b).

## DISCUSSION

This is, to our knowledge, the first real‐world study investigating the survival benefit of CLND versus LNE in melanoma patients with lymph node macrometastasis receiving postoperative adjuvant systemic therapy. We observed a preference for CLND in patients with AJCCv8 stages IIIC and IIID, compared to patients with stage IIIB disease. RFS and OS were comparable between the two groups, while CLND was associated with prolonged NFS. Multivariate Cox regression analysis adjusting for more than three lymph nodes and the duration of adjuvant systemic therapy, however, showed a significant prolongation of RFS by CLND in comparison to LNE. Interestingly, CLND did not have a significant impact on OS. On the other hand, patients undergoing CLND experienced a higher incidence of complications, particularly chronic lymphedema.

Comparable to the results in patients with a positive Sentinel lymph node, there was no overall survival benefit by CLND compared to LNE only in this retrospective analysis. However, NFS was improved in the CLND group. Of all 320 patients with lymph node macrometastasis, most patients (n = 222; 69.4%) received CLND. Due to the retrospective nature of the study, a large heterogeneity between the CLND and the LNE group was observed. In particular, the number of histologically confirmed lymph node metastases and associated stages of disease differed between the two groups. Since the number of lymph node metastases decisively influences survival,^18^, ^19^, ^21^, ^28^, ^29^ the effects of the intervention were difficult to decipher within the overall study cohort. After adjusting for patients with more than three positive lymph nodes and the duration of adjuvant systemic therapy in multivariate Cox proportional hazards regression analysis, CLND showed improved RFS over LNE. Concerning the type of progression, fewer regional lymphatic metastasis recurrences were detected in the CLND group, while distant metastasis and in‐transit‐metastasis formation as well as local recurrence in the area of the primary tumor were similar between the groups. These findings suggest that the performance of CLND may be justified in a subset of patients with lymph node macrometastasis, despite the higher rate of postoperative complications. It is likely that this effect is particularly relevant in patients who do not receive adjuvant radiation therapy, which could also reduce the risk of local recurrence.^30^, ^31^, ^32^ Of note, adjuvant radiation therapy was associated with worse RFS and OS in the univariate Cox‐regression models of this study. This may be attributable to the fact that adjuvant radiation therapy is recommended when independent risk factors such as more than three metastatic lymph nodes, a maximum lymph node diameter greater than 4 cm, and extracapsular spread are present.^30^

In contrast to RFS, multivariate Cox proportional hazards regression analysis for OS did not reveal any survival benefit for CLND over LNE. This may be explained by the local benefits of the CLND mentioned above. On the other hand, further melanoma‐specific factors such as subsequent therapies and non‐melanoma‐specific factors substantially influence the OS, which make it a relatively unspecific outcome parameter that is often complemented by melanoma‐specific survival. Furthermore, despite possible clinical benefits the relatively small study population and the rather short follow‐up time may have been insufficient to detect significant effects on OS.

The study revealed that upon relapse after LNE second‐line CLND was only performed in a small subset of patients. Consequently, it was not possible to assess the therapeutic benefit of second‐line CLND.

CLND was associated with a higher rate of complications compared to LNE. In particular, lymphedema was a more frequent complication in CLND. As expected from other studies,^33^, ^34^ a higher rate of complications was observed for inguinal interventions compared to axillary or cervical interventions. Due to a higher percentage of inguinal interventions in the LNE group, it can be assumed that the overall complication rate for the LNE group may be overestimated in comparison to the CLND group and differences in complication rates may actually be even larger after adjusting for the location of the interventions.

Given differences in the distribution of the locations of the interventions and the observed tendency to recommend CLND in patients with more than one clinically suspected lymph node macrometastasis, retrospective analyses may not be the most suitable approach. Accordingly, prospective clinical studies would be required to provide a thorough insight on the role of CLND compared to LNE in patients receiving consecutive adjuvant therapy.

We analyzed the effects of CLND or LNE in patients with lymph node macrometastasis who subsequently received an adjuvant systemic therapy. With the approval of neoadjuvant treatment regimens,^7^, ^8^, ^9^ however, the surgical procedure may need to be adapted. Whereas the type and extent of surgery could be chosen individually in the NADINA and SWOG 1801 trials,^7^, ^8^ only the index lymph node was removed in the PRADO extension cohort of the OpACIN‐neo trial.^9^ As observed in this study, omission of CLND in the PRADO extension cohort of the OpACIN‐neo trial^9^ resulted in significantly less surgical morbidity compared to the excision of the index lymph node only. The findings of our study, showing a benefit in RFS for CLND over LNE in melanoma patients under adjuvant systemic therapy, emphasize that a careful evaluation of the surgical procedure may also be required for neoadjuvant treatment regimens. Especially in patients with no pathological response, who account for about one fourth of all patients,^7^ CLND may still be a valuable treatment option. Therefore, suitable biomarkers predicting the response to immunotherapies would also serve to identify patients who would benefit from additional CLND, even in the age of neoadjuvant treatment.

## CONCLUSIONS

This study compared CLND and selective excision of suspected lymph nodes only. CLND showed improved NFS. After adjusting for number of positive lymph nodes and duration of adjuvant systemic therapy, CLND was associated with significantly less relapses, while OS was not significantly influenced. Meanwhile, the number of complications was significantly higher in the CLND group.

In the setting of adjuvant systemic therapies, these findings support the performance of CLND to achieve improved local control. However, due to the higher number of complications, individual patient characteristics such as age or general condition should be included in the decision favoring a personalized approach on choosing the extent of surgical procedures in patients with nodal macrometastasis. This approach should be reconsidered after the approval of neoadjuvant regimens in the future.

## CONFLICT OF INTEREST STATEMENT

K.E.M. received travel support from Novartis and was a member of the advisory board of Imnotech GmbH, which holds patents on an anti‐CD2 and an anti‐CD7 single‐domain antibody; J.C.H. declares honoraria from Amgen, Bristol Myers Squibb, Delcath, GlaxoSmithKline, Immunocore, Merck Sharp & Dohme, Novartis, Pierre Fabre, Sanofi, and Sun Pharma, outside the submitted work; J.S. declares no conflicts of interest; M.E. declares honoraria and travel support from Bristol Myers Squibb, Immunocore, Novartis, and Pierre Fabre, outside the submitted work; M.A.S. declares honoraria for consulting and/or speaker's fees and/or travel support from Bristol Myers Squibb, Immunocore, Kyowa Kirin, Merck Sharp & Dohme, Novartis, Pierre Fabre, Sanofi, Regeneron, Sun Pharma, and Takeda; J.O. declares no conflicts of interest; A.T. received research funding from Almirall Hermal and consulting and/or speaker's honoraria and/or travel support from Almirall Hermal, Bristol Myers Squibb, Boehringer Ingelheim, GlaxoSmithKline, Janssen, Kyowa Kirin, Merck Sharp & Dohme, Pfizer, Pierre Fabre, Recordati Rare Diseases, and Sanofi; D.T. reports consultancy, speaker fees, or travel grants from Bristol Myers Squibb, Roche, Novartis, Sanofi, Recordati, Kyowa Kirin, Sun Pharma, and Pierre Fabre; J.K. declares no conflicts of interest; M.S. received consulting fees and travel support from Bristol Myers Squibb, Merck Sharp & Dohme, Kyowa Kirin, Novartis, Pierre Fabre, Sun Pharma, and Immunocore; C.B. declares honoraria for consulting and/or speaker's fees from Almirall Hermal, Bristol Myers Squibb, Novartis, Merck Sharp & Dohme, Immunocore, Delcath, InflaRx, Pierre Fabre, Sanofi, and Regeneron, outside the submitted work; C.P. received honoraria and travel support from AbbVie, Bristol Myers Squibb, Pelpharma, Novartis, Leo Pharma, Galderma, Celgene, Iovance, Sun Pharma, Sanofi Genzyme, Merck Sharp & Dohme, Merck, Almirall, Pfizer, Pierre Fabre, DSD, AstraZeneca, Eli Lilly, Janssen, Takeda, and Amazentis, all unrelated to this work; T.B. received grants from Almirall, Celgene‐BMS, Lilly, Novartis, Sanofi Genzyme, and Regeneron; consulting fees from AbbVie, Alk‐Abello, Almirall, Boehringer Ingelheim, Leo Pharma, Lilly, Novartis, Sanofi Genzyme, and Viatris; honoraria from Alk‐Abello, Almirall, GSK, Leo Pharma, Lilly, Novartis, Sanofi Genzyme, and Regeneron; and is a member of the advisory board of Alk‐Abello, Almirall, Boehringer Ingelheim, Leo Pharma, Lilly, Novartis, Sanofi Genzyme, and Viatris; O.D.P. declares honoraria and travel support from Merck Sharp & Dohme, Kyowa Kirin, Pierre Fabre, Sanofi, Sun Pharma, and Bristol Myers Squibb.

## Supporting information



Supplementary information

## References

[1] Helvind NM , Brinch‐Moller Weitemeyer M , Chakera AH , et al. Stage‐Specific Risk of Recurrence and Death From Melanoma in Denmark, 2008‐2021: A National Observational Cohort Study of 25 720 Patients With Stage IA to IV Melanoma. JAMA Dermatol. 2023;159:1213‐1222. 37650576 10.1001/jamadermatol.2023.3256 PMC10472263

[2] Eggermont AMM , Kicinski M , Blank CU , et al. Five‐Year Analysis of Adjuvant Pembrolizumab or Placebo in Stage III Melanoma. NEJM Evid. 2022;1:EVIDoa2200214. 10.1056/EVIDoa2200214 38319852

[3] Grossmann KF , Othus M , Patel SP , et al. Adjuvant Pembrolizumab versus IFNalpha2b or Ipilimumab in Resected High‐Risk Melanoma. Cancer Discov. 2022;12:644‐653. 34764195 10.1158/2159-8290.CD-21-1141 PMC8904282

[4] Larkin J , Del Vecchio M , Mandala M , et al. Adjuvant Nivolumab versus Ipilimumab in Resected Stage III/IV Melanoma: 5‐Year Efficacy and Biomarker Results from CheckMate 238. Clin Cancer Res. 2023;29:3352‐3361. 37058595 10.1158/1078-0432.CCR-22-3145 PMC10472092

[5] Dummer R , Hauschild A , Santinami M , et al. Five‐Year Analysis of Adjuvant Dabrafenib plus Trametinib in Stage III Melanoma. N Engl J Med. 2020;383:1139‐1148. 32877599 10.1056/NEJMoa2005493

[6] Maio M , Lewis K , Demidov L , et al. Adjuvant vemurafenib in resected, BRAF(V600) mutation‐positive melanoma (BRIM8): a randomised, double‐blind, placebo‐controlled, multicentre, phase 3 trial. Lancet Oncol. 2018;19:510‐520. 29477665 10.1016/S1470-2045(18)30106-2

[7] Blank CU , Lucas MW , Scolyer RA , et al. Neoadjuvant Nivolumab and Ipilimumab in Resectable Stage III Melanoma. N Engl J Med. 2024;391:1696‐1708. 38828984 10.1056/NEJMoa2402604

[8] Patel SP , Othus M , Chen Y , et al. Neoadjuvant‐Adjuvant or Adjuvant‐Only Pembrolizumab in Advanced Melanoma. N Engl J Med. 2023;388:813‐823. 36856617 10.1056/NEJMoa2211437 PMC10410527

[9] Reijers ILM , Menzies AM , van Akkooi ACJ , et al. Personalized response‐directed surgery and adjuvant therapy after neoadjuvant ipilimumab and nivolumab in high‐risk stage III melanoma: the PRADO trial. Nat Med. 2022;28:1178‐1188. 35661157 10.1038/s41591-022-01851-x

[10] Leiter U , Stadler R , Mauch C , et al. Final Analysis of DeCOG‐SLT Trial: No Survival Benefit for Complete Lymph Node Dissection in Patients With Melanoma With Positive Sentinel Node. J Clin Oncol. 2019;37:3000‐3008. 31557067 10.1200/JCO.18.02306

[11] Faries MB , Thompson JF , Cochran AJ , et al. Completion Dissection or Observation for Sentinel‐Node Metastasis in Melanoma. N Engl J Med. 2017;376:2211‐2222. 28591523 10.1056/NEJMoa1613210 PMC5548388

[12] Brown RE , Ross MI , Edwards MJ , et al. The prognostic significance of nonsentinel lymph node metastasis in melanoma. Ann Surg Oncol. 2010; 17: 3330‐3335. 20645010 10.1245/s10434-010-1208-8

[13] Susok L , Nick C , Becker JC , et al. Waiving Subsequent Complete Lymph Node Dissection in Melanoma Patients with Positive Sentinel Lymph Node Does Not Result in Worse Outcome on 20‐Year Analysis. Cancers (Basel). 2021; 13(21):5425. 34771588 10.3390/cancers13215425 PMC8582468

[14] Angeles CV , Kang R , Shirai K , Wong SL . Meta‐analysis of completion lymph node dissection in sentinel lymph node‐positive melanoma. Br J Surg. 2019;106:672‐681. 30912591 10.1002/bjs.11149

[15] Boada A , Tejera‐Vaquerizo A , Ribero S , et al. Factors associated with sentinel lymph node status and prognostic role of completion lymph node dissection for thick melanoma. Eur J Surg Oncol. 2020;46:263‐271. 31594672 10.1016/j.ejso.2019.09.189

[16] Baecher H , Gerken M , Knoedler L , et al. Complete lymph node dissection in cutaneous melanoma patients with positive sentinel lymph node: Outcome and predictors in a retrospective cohort study over 16 years. J Plast Reconstr Aesthet Surg. 2024;92:33‐47. 38489985 10.1016/j.bjps.2024.02.056

[17] Persa OD , Knuever J , Mauch C , Schlaak M . Complete lymph node dissection or observation in melanoma patients with multiple positive sentinel lymph nodes: A single‐center retrospective analysis. J Dermatol. 2018;45:1191‐1194. 30095864 10.1111/1346-8138.14577

[18] Morton DL , Wanek L , Nizze JA , et al. Improved long‐term survival after lymphadenectomy of melanoma metastatic to regional nodes. Analysis of prognostic factors in 1134 patients from the John Wayne Cancer Clinic. Ann Surg. 1991; 214: 491‐499; discussion 499‐501. 1953101 10.1097/00000658-199110000-00013 PMC1358554

[19] Coit DG , Rogatko A , Brennan MF . Prognostic factors in patients with melanoma metastatic to axillary or inguinal lymph nodes. A multivariate analysis. Ann Surg. 1991;214:627‐636. 1953117 10.1097/00000658-199111000-00014 PMC1358620

[20] Karakousis CP , Emrich LJ , Driscoll DL , Rao U . Survival after groin dissection for malignant melanoma. Surgery. 1991;109:119‐126. 1992543

[21] White RR , Stanley WE , Johnson JL , et al. Long‐term survival in 2,505 patients with melanoma with regional lymph node metastasis. Ann Surg. 2002; 235: 879‐887. 12035046 10.1097/00000658-200206000-00017 PMC1422519

[22] Rossi CR , Mozzillo N , Maurichi A , et al. The number of excised lymph nodes is associated with survival of melanoma patients with lymph node metastasis. Ann Oncol. 2014;25:240‐246. 24356635 10.1093/annonc/mdt510

[23] Chan AD , Essner R , Wanek LA , Morton DL . Judging the therapeutic value of lymph node dissections for melanoma. J Am Coll Surg. 2000;191:16‐22; discussion 22‐23. 10898179 10.1016/s1072-7515(00)00313-6

[24] Clark T , Parekh DJ , Cookson MS , et al. Randomized prospective evaluation of extended versus limited lymph node dissection in patients with clinically localized prostate cancer. J Urol. 2003;169:145‐147; discussion 7‐8. 12478123 10.1016/S0022-5347(05)64055-4

[25] Wilke LG , McCall LM , Posther KE , et al. Surgical complications associated with sentinel lymph node biopsy: results from a prospective international cooperative group trial. Ann Surg Oncol. 2006;13:491‐500. 16514477 10.1245/ASO.2006.05.013

[26] Verver D , Madu MF , Oude Ophuis CMC , et al. Optimal extent of completion lymphadenectomy for patients with melanoma and a positive sentinel node in the groin. Br J Surg. 2018;105:96‐105. 29095479 10.1002/bjs.10644 PMC5765473

[27] Henderson MA , Gyorki D , Burmeister BH , et al. Inguinal and Ilio‐inguinal Lymphadenectomy in Management of Palpable Melanoma Lymph Node Metastasis: A Long‐Term Prospective Evaluation of Morbidity and Quality of Life. Ann Surg Oncol. 2019;26:4663‐4672. 31515719 10.1245/s10434-019-07810-0

[28] Moreau S , Saiag P , Aegerter P , et al. Prognostic value of BRAF(V(6)(0)(0)) mutations in melanoma patients after resection of metastatic lymph nodes. Ann Surg Oncol. 2012;19:4314‐4321. 22772867 10.1245/s10434-012-2457-5

[29] Mosquera C , Vora HS , Vohra N , Fitzgerald TL . Population‐Based Analysis of Completion Lymphadenectomy in Intermediate‐Thickness Melanoma. Ann Surg Oncol. 2017;24:127‐134. 27464609 10.1245/s10434-016-5460-4

[30] Henderson MA , Burmeister BH , Ainslie J , et al. Adjuvant lymph‐node field radiotherapy versus observation only in patients with melanoma at high risk of further lymph‐node field relapse after lymphadenectomy (ANZMTG 01.02/TROG 02.01): 6‐year follow‐up of a phase 3, randomised controlled trial. Lancet Oncol. 2015;16:1049‐1060. 26206146 10.1016/S1470-2045(15)00187-4

[31] Agrawal S , Kane JM, 3rd , Guadagnolo BA , et al. The benefits of adjuvant radiation therapy after therapeutic lymphadenectomy for clinically advanced, high‐risk, lymph node‐metastatic melanoma. Cancer. 2009;115:5836‐5844. 19701906 10.1002/cncr.24627

[32] Lee RJ , Gibbs JF , Proulx GM , et al. Nodal basin recurrence following lymph node dissection for melanoma: implications for adjuvant radiotherapy. Int J Radiat Oncol Biol Phys. 2000;46:467‐474. 10661355 10.1016/s0360-3016(99)00431-9

[33] Friedman JF , Sunkara B , Jehnsen JS , et al. Risk factors associated with lymphedema after lymph node dissection in melanoma patients. Am J Surg. 2015;210:1178‐1184; discussion 84. 26482511 10.1016/j.amjsurg.2015.08.014

[34] Deban M , Vallance P , Jost E , et al. Higher Rate of Lymphedema with Inguinal versus Axillary Complete Lymph Node Dissection for Melanoma: A Potential Target for Immediate Lymphatic Reconstruction? Curr Oncol. 2022;29:5655‐5663. 36005184 10.3390/curroncol29080446 PMC9406378

