# Supplementary material for: Complete lymph node dissection versus selective lymph node extirpation in melanoma patients with nodal macrometastasis and adjuvant systemic therapy
Source: J Dtsch Dermatol Ges. 2026 Jan 21;24(8):1052–61. doi: 10.1111/ddg.15967 (PMC13435047; doi:10.1111/ddg.15967)
Supplement: Supplementary file 1 [file DDG-24-1052-s001.docx]

[[ONLINE-SUPPLEMENT]]

TABLE S1 Long-term outcomes after complete lymph node dissection (CLND) vs. lymph node extirpation (LNE) of suspect lymph nodes only.

[[Online Supplement]]

| **Table S1** | | |
| --- | --- | --- |
|  | **CLND (n = 222; 69.4%)** | **LNE (n = 98; 30.6%)** |
| ***Progression*** |  | |
| Yes | 98 (44.1%) | 51 (52.0%) |
| No | 124 (55.9%) | 47 (48.0%) |
| ***Type of progression*** |  |  |
| Regional lymphatic metastasis | 16 (16.3%) | 17 (33.3%) |
| Distant metastasis | 74 (75.5%) | 36 (70.6%) |
| In-transit-metastasis or local recurrence | 11 (11.2%) | 7 (13.7%) |
| ***Death*** |  | |
| Yes | 42 (18.9%) | 22 (22.4%) |
| No | 180 (81.1%) | 76 (77.6%) |
| ***Recurrence-free survival (RFS)*** |  |  |
| Median in months | 43 | 30 |
| ***Nodal metastasis-free survival (NFS)*** |  |  |
| Median in months | not reached | not reached |
| ***Overall survival (OS)*** |  |  |
| Median in months | not reached | not reached |

*Abbr.:* CLND, complete lymph node dissection; LNE, lymph node extirpation; NFS, nodal metastasis-free survival; OS, overall survival; RFS, recurrence-free survival.

TABLE S2 Complications after CLND vs. LNE.

| **Table S2** | | | | | | | | |  | |  |
| --- | --- | --- | --- | --- | --- | --- | --- | --- | --- | --- | --- |
|  | **CLND (n = 222)** | | | | **LNE (n = 98)** | | | | | | |
| ***Complication after CLND or LNE*** | ***Total (n = 222)*** | ***Inguinal (n = 64)*** | ***Axillary (n = 114)*** | ***Cervical (n = 44)*** | ***Total (n = 98)*** | ***Inguinal (n = 40)*** | ***Axillary (n = 31)*** | ***Cervical (n = 19)*** | | ***Other (n = 8)*** | |
| Yes | 84 (37.8%) | 31 (48.4%) | 42 (36.8%) | 11 (25.0%) | 20 (20.4%) | 13 (32.5%) | 5 (16.1%) | 2 (10.5%) | | 0 | |
| No | 129 (58.1%) | 30 (46.9%) | 69 (60.5%) | 30 (68.2%) | 72 (73.5%) | 24 (60.0%) | 25 (80.6%) | 17 (89.5%) | | 6 (75.0%) | |
| Unknown | 9 (4.1%) | 3 (4.7%) | 3 (2.6%) | 3 (6.8%) | 6 (6.1%) | 3 (7.5%) | 1 (3.2%) | 0 | | 2 (25.0%) | |
| ***Type of complication after CLND or LNE*** | ***Total (n = 89)*** | ***Inguinal (n = 32)*** | ***Axillary (n = 46)*** | ***Cervical (n = 11)*** | ***Total (n = 20)*** | ***Inguinal (n = 13)*** | ***Axillary (n = 5)*** | ***Cervical (n = 2)*** | | ***Other (n = 0)*** | |
| Seroma/lymphocele | 35 (39.3%) | 11 (34.4%) | 21 (45.7%) | 3 (27.3%) | 12 (60.0%) | 8 (61.5%) | 4 (80.0%) | 0 | | 0 | |
| Lymphatic fistula | 5 (5.6%) | 3 (9.4%) | 2 (4.3%) | 0 | 0 | 0 | 0 | 0 | | 0 | |
| Bleeding/hematoma | 7 (7.9%) | 4 (12.5%) | 2 (4.3%) | 1 (9.1%) | 2 (10.0%) | 1 (7.7%) | 0 | 1 (50.0%) | | 0 | |
| Lymphedema | 38 (42.7%) | 14 (43.8%) | 20 (43.5%) | 4 (36.4%) | 4 (20.0%) | 3 (23.1%) | 1 (20.0%) | 0 | | 0 | |
| Facial nerve paresis | 3 (3.3%) | – | – | 3 (27.3%) | 1 (5.0%) | – | – | 1 (50.0%) | | 0 | |
| Infection | 1 (1.1%) | 0 | 1 (2.2%) | 0 | 1 (5.0%) | 1 (7.7%) | 0 | 0 | | 0 | |

*Abbr.:* CLND, complete lymph node dissection; LNE, lymph node extirpation
